# Assessing attitudes toward LGB people in young adolescents

**DOI:** 10.1371/journal.pone.0305057

**Published:** 2024-10-08

**Authors:** Elise Stiekema, Matthijs J. Warrens, Diana D. van Bergen, Sanne Parlevliet

**Affiliations:** Department of Education, University of Groningen, Groningen, The Netherlands; University of Technology Sydney, AUSTRALIA

## Abstract

Since people’s attitude toward lesbians, gay men and bisexual people (LGB) affects young LGB people’s mental health and subsequently their quality of life, it is important to establish people’s attitudes toward LGB people. The current study examined attitudes toward lesbian, gay and bisexual people among Dutch students and the psychometrical characteristics of adapted instruments measuring attitudes toward sexual diversity. The students in the sample (N = 1.633) were recruited from secondary schools. The participants completed questionnaires with scales measuring attitudes toward LGB people. The results indicate that participants hold positive attitudes overall, but there are differences with regard to gender and educational level. In addition, the used instruments prove to be psychometrically sound for measuring LGB attitudes and this work contributes to the empirical debate about whether adolescents’ attitudes toward lesbians, gay men and bisexual people underly domains of a general attitude toward LGB people. Our results indicate that students do not seem to distinguish among sexually diverse subgroups much, possibly a sign of increased awareness and knowledge of LGB groups. The use of one general measure of attitudes of sexually diverse people may be considered in future research.

## Introduction

Lesbian, gay and bisexual (LGB) young people have a higher prevalence of psychological issues and psychiatric disorders than heterosexual young people. For example, they report elevated levels of psychological distress [1], and they more frequently report depression and suicidal thoughts than their heterosexual peers [2]. Lesbian, gay and bisexual youth also report lower psychological wellbeing and social support [3–5]. These disparities in mental health [6] are related to the prejudice and stigmatization that LGB young people experience [7, 8]. LGB young adolescents report to especially experience this in the school context [9]. In addition, among young adolescents, schools are important social contexts that contribute to develop mental and health outcomes [10]. For sexual minority youths in particular, the social context of schools can promote both vulnerability and resilience [11, 12]. Thus, important correlates for the increased propensity to mental health issues are their peers’ attitudes toward sexual diversity [13–15].

Since peer attitudes toward sexual diversity can affect LGB people’s wellbeing [2, 7, 8], it is important to establish how young adolescents think about and feel toward LGB people. Past research indicates that people differ in their attitudes toward lesbians as opposed to gay men. This is related to societal norms about masculinity and femineity. If people, and especially males, reject gay men this is often due to stereotyping as they deviate from masculine behavioral norms [16–19]. Women are less likely to be influenced by this tendency and attitudes toward lesbians are generally more positive in comparison to attitudes toward gay men [20]. Therefore, it seems appropriate not to measure LGB people as one stigmatized group but to use separate measures [16–18, 21, 22]. However, these findings on attitudes toward various sexual minority groups are rather dated, and more recent research indicates that, at present, some sexual minority youth groups adopt fluid and alternative labels of sexuality rather than L, G or B [23]. This raises the question whether the differentiation into three subgroups, namely lesbian, gay and bisexual is adequate when measuring current attitudes toward sexual diversity in society, or that at present, it would make more sense to measure attitudes toward sexual diversity with constructs that match newly developed (broader) labels.

Demographics and socio-cultural constructs seem to influence how people think about lesbians, gay and bisexual people. As mentioned, gender in particular seems to play a predictive role to sexual diversity attitudes [20, 24–26]. In addition, whether educational level influences young adolescents’ attitudes toward LGB people, has not been studied comprehensively but this factor may be relevant. Studies of adult populations have found that having accomplished a high level of education is associated with more accepting attitudes of sexual diversity [27, 28]. Thus, it seems theoretically plausible that education level might also influence youths’ attitudes toward LGB people.

### Context of the study

The present study was performed in the Netherlands, which is an interesting case with respect to LGB acceptance. It is a liberal Western European country, with relatively high acceptance rates of sexual diversity. For example, in 2001, the Netherlands was the first country to introduce and legalize gay marriage [29]. Although the attitudes of Dutch adults as well as Dutch youth toward LGB people have become even more positive over the years [24, 30], two important comments should be made. First, only minor improvements in the psychological wellbeing of Dutch LGB adolescents have been noted, and they continue to experience a lower self-reported quality of life and more stressors, for example, twice the bullying rates of heterosexual adolescents. Second, in early adolescence (12 to 14 years), youth in the Netherlands, as in other Western countries [31], continue to have more negative attitudes toward LGB people than in mid- (15 to 17 years) or late adolescence (18 to 20 years) [30]. Hence, a particular challenge thus seems to lie in enhancing LGB attitudes among young adolescents. Additionally, there are also a number of methodological considerations to the findings about attitude of sexual diversity among Dutch youth. Outcomes are based on just a few single survey items [27], while more extensive measures using scales is considered to be more reliable. However, in the Netherlands, there are few instruments for assessing attitudes and intervening behavior, and their reliability and validity for use with Dutch young adolescents have not been established.

Since 2012 Dutch schools are obliged by national law to provide students with education on sexual diversity, and explicitly teach respect for the topic, as a part of citizenship education (goal 43, SLO, 2016). Schools can decide to what extent and in what way they implement sexual diversity education in their curriculum, as long as it is affirmative in nature. In the Netherlands there are multiple educational levels. Most youth enter secondary education when they are 12 years old (comparable to grade 7). There are roughly three educational levels/tracks to distinguish in secondary schools: pre-vocational education, senior general secondary education (prior training for higher professional education) and pre-university education.

In order to better understand the association between LGB young people’s disadvantaged mental health and attitudes toward LGB young people, especially among the younger age groups, researchers require quantitative instruments for assessing these constructs with high reliability and validity. The present study, examines whether young adolescents hold positive attitudes of LGB of LGB people when using scales rather than single items, and investigates if gender and educational track (level) moderates these attitudes. For this reason, we also test the validity and reliability of various adapted existing instruments of LGB attitudes in order to see if they can, in present times, (still) accurately measure (early) adolescents’ attitudes toward LGB people [32–34]. For this study, the following scales were selected by three LGB experts (redacted for review) in the Dutch context, following their hypothesized suitability in a Dutch context: The Attitudes Toward Lesbians and Gay Men scale [32] and the Attitudes Toward Bisexual People scale [34]. To date, the psychometric properties (e.g., factorial validity and reliability) of these scales have not been thoroughly investigated across cultures.

The purpose of the current study is as follows: (a) To test the factorial validity and reliability of the adapted instruments regarding measuring attitudes toward lesbians, gay men and bisexual people for the target group; (b) to test the measurement invariance of the adapted instruments across gender and educational level; (c) to examine attitudes toward lesbians, gay men and bisexual people among Dutch early adolescents (eighth- and ninth-graders with gender and educational track as moderators); (d) to explore relationships between attitudes toward LGB people (i.e., does being negative (or positive) to gay men also means a negative (or positive) attitude to lesbians?); and (e) to contribute to the empirical debate about whether adolescents’ attitudes toward lesbians, gay men and bisexual people are underlying domains of a general attitude toward LGB people.

### Theoretical framework

#### Research on instruments that measure LGB attitudes

Previous studies examining attitudes toward sexual diversity, particularly among lesbian, gay and bisexual individuals, have employed various instruments to measure these attitudes. One common approach has been the utilization of a single instrument to assess attitudes toward homosexuality or sexual minorities as a whole. These instruments often included items that gauge both positive and negative attitudes toward LGB people, providing a broad understanding of attitudes toward sexual minorities [35]. However, bisexual people as a minority group are often not included in studies about sexual diversity. Yet, in absolute numbers this group is actually larger than the group constituting gay and lesbian people [36]. Also, the well-being of bisexual students is significantly lower than that of lesbian and gay male students in the Netherlands and in the USA [37, 38] since they tend to experience double stigma. Biphobia is a psychological construct that consists of negative attitudes about bisexuality and bisexual people [39], which can be present in heterosexual as well as lesbian or gay individuals [40].

In addition, researchers have also recognized the unique experiences and stereotypes associated with different sexual orientations. As such, some studies have adopted a more nuanced approach by employing separate measures for attitudes toward lesbians and gay men. This approach acknowledges the distinct social perceptions and prejudices that exist within society, allowing for a more detailed examination of attitudes toward specific LGB subgroups [41]. One notable example of this is the work of Herek, who developed the Attitudes Toward Lesbians and Gay Men Scale [32], a widely used and longstanding measure of people’s attitudes toward LGB people. Herek [21, 32, 41, 42] has shown that this instrument is psychometrically sound for assessing people’s attitudes toward lesbians and gay men in the United States, but it has also been used more recently in various countries and contexts, albeit in slightly modified versions (e.g., the United Kingdom, Canada and Brazil). However, these recently modified versions have not been psychometrically tested [43–47]. The instrument is comprised of two subscales, Attitudes Toward Lesbians and Attitudes Toward Gay Men, which is important, as Herek [32] demonstrated that US adults differentiate in what they think about lesbians and gay men. Subsequently, Van de Meerendonk et al. [33] conducted a representative test on the applicability of the slightly adjusted and translated scale in the Netherlands, and found support for the scale’s acceptable reliability and construct and discriminant validity among Dutch adults. However, Herek’s scale [32] was tested more than 20 years ago, and has never been validated among Dutch adolescents in particular. The current study fills this gap.

Moreover, bisexual people were not addressed by Herek. The current study therefore added a third subscale to be investigated for its psychometric properties, the Attitude Toward Bisexual People scale. A study based on the subscale by Mulick and Wright [34] has provided empirical support for the existence of the construct of biphobia and suggests that it exists in both the heterosexual and homosexual communities. The biphobia instrument is an adapted instrument of existing scales which measure constructs believed to be similar to biphobia, such as homophobia [35, 48] and AIDS phobia [49]. The instrument proved to be accurate in measuring biphobia in a convenience sample of undergraduate students from a large Mid-Western university in the USA [34], and has been used in different cultural contexts [50, 51].

### Moderators in attitudes toward LGB people

There are different social and demographic differences or moderators visible in regard to people’s attitudes toward sexual diversity [52]. We will focus on gender and educational level as potential effects in regard to attitude. These moderators will be discussed next:

#### Gender norms and gender identity

Whether one maintains stereotypical gender norms can influence attitudes toward LGB individuals [20, 53, 54]. Gender norms (binary norms) are culturally shared expectations about the characteristics a boy/man or girl/woman should possess and how they should behave (i.e., gender conformity) [20, 55]. These gender norms tend to be oppositional for men and women, for example, women in Western societies tend to be expected to be sensitive and caring, whereas men tend to be expected to be more dominant and assertive. The extent to which traditional gender norms or beliefs exist in an individual have been found to be relevant to their attitude toward gay men [20, 24, 56]. The stereotyping of gay men and the increased importance that males attach to gender conformity in males lead to a greater propensity to reject gay men [16–19]. Women however, seem to be less likely to be influenced by a preference for “gender conformity” in regard to gay men than males [20]. There are multiple theories supporting these socially set behavior norms for men and women as well as how sexual socialization influences attitudes toward non-heterosexuality, among which the gender conflict theory [57, 58].

Gender identity may also influence people’s attitudes toward lesbian women versus gay men. Possibly due to the sexualization (‘eroticization’) of lesbian women and gender norms that particularly bias gay men, general attitudes toward gay men tend to be significantly more negative than attitudes toward lesbians in male adolescents, at least in Western contexts [20, 22, 24–26]. Therefore, our assumption is that it seems useful to distinguish two target groups, lesbians and gay men, rather than an overarching category of “homosexuals” when examining attitudes toward sexual minorities [17, 20, 24, 54, 59]. Nevertheless, since the majority of studies that have measured distinct attitudes toward gay and lesbian persons are somewhat outdated, and young people today may have arrived at new perceptions of sexual diversity due to social and cultural changes, it is important to examine this assumption. This study aims to fill this gap.

#### Educational level

Studies among adults show that enrollment in higher levels of education are associated with more affirmative and inclusive attitudes toward sexual diversity [28, 60]. Even though young adolescents’ attitudes toward LGB people have not been studied comprehensively across different educational levels, it might have a substantial effect. Dutch adolescents in lower tracks (pre-vocational education) indicate to feel less safe in school as their peers in higher educational levels (e.g., pre-academic tracks). In addition, young LGB adolescents in pre-vocational education seem more prone to become victims of bullying than in pre-academic tracks as they report more victimization in school due to their sexual orientation [61]. Therefore, the present study will examine variance in attitudes toward LGB people across different educational levels.

## Method

### Procedure and samples

This study is part of a larger project concerning a reading intervention for young adolescents on sexual diversity in Dutch language and literature classes. We searched for contact details of schools and teachers on the internet, with an equal division across all provinces of the Netherlands. In the Netherlands, there are between 1,400 and 1,500 secondary schools. For this study, we reached out to approximately 100 schools across all regions throughout the country. Teachers and schools were sent an informational email about the project and corresponding research. Furthermore, an advertisement was posted on a national Facebook group page for Dutch language and literature teachers. Teachers who found the project through our call on Facebook, as well as the teachers we reached out to via their websites, self-registered for the study once their schoolboard had approved their participation. Participating students completed a digital survey in the classroom under their teachers’ supervision. Both students and parents filled out the informed consent forms prior to participating in the survey. For this study, we use two separate samples since it concerns two groups of participants that completed the survey in two different years. During the data collection, schools had to take the COVID pandemic and constantly changing rules and policies into account, e.g., partially/temporarily closing of schools, absence of affected students, strict rules of conduct within classrooms limiting interpersonal contact. Because of the societal differences between the two periods the two samples were analyzed separately. The study was approved by the Ethics Committee of the Faculty of Behavioral and Social Sciences of the University of Groningen.

#### Sample 1

The first sample consisted of 742 students in the eighth or ninth grade. 850 participants started the questionnaire. The records of 108 participants were removed because they completed none or only the first few questions. There were no further missing data. Most of the 742 students that completed the full survey did this in October-November 2020; some completed the survey in March 2021. The participants came from 39 classes at 12 different schools. The age range varied from 12 to 16 years old, with an average of 13.6 years (SD = .81). The sample consisted of 399 girls (53.8%), 329 boys (44.3), two respondents who were assigned male at birth but identify as girls (0.3%) and 11 students who identify as neither boy nor girl (1.5%). There were no students that were assigned female at birth but identify as boys. Furthermore, 124 students followed pre-vocational education (15.8%), 227 students followed senior general secondary education (30.6%) and 390 students were in pre-university education (52.6%). In addition, 27.0% of the students indicated that they have a religion (the degree of active practice is unclear).

#### Sample 2

The second sample consisted of 892 students from 48 classes of 12 schools and that were in the eighth or ninth grade. 950 participants started the questionnaire, but the records of 58 participants were removed because they completed none or only the first few questions. There were no further missing data. These participants completed the same survey as sample 1, but with additional questions related to topics on LGB bullying and intervening behavior added at the end. This sample completed the survey in October-November 2021. The age range varied from 12 to 16 years old, with an average of 13.7 years (SD = .77). The sample consisted of 404 girls (45.3%), 433 boys (48.5%), one student who was assigned male at birth but identifies as a girl (0.1%), two students who were assigned female at birth but identify as boys (0.2%), 13 students who identified as neither boy nor girl (1.5%), 23 students who indicated that they are not sure (2.6%) about their gender (identity) and 16 students stated that they identified otherwise (1.8%). This latter option was not included for sample 1, but was added after feedback from students.

Furthermore, 286 students followed pre-vocational education (32.0%), 229 students were enrolled in senior general secondary education (25.7%) and 377 students were in pre-university tracks (42.3%). Only 23.1% of the students stated to be religious (the degree of active practice is unclear), which is somewhat lower compared to the national average. For both samples, the location of the participating schools in urban versus rural regions, as well as their size was fairly comparable to national statistics of this aspect (Statistics Netherlands, CBS, 2021). In addition, about 20% of adolescents in the Netherlands have a non-Western migration background. In our samples this applied to, respectively, 20.5% and 17.4% of the participants.

### Measurements and analysis plan

#### Attitudes toward lesbians and gay men

To assess students’ attitudes toward lesbians and gay men, we used two subscales derived from Van de Meerendonk et al.’s [33] study on attitudes toward lesbians and gay men in the Netherlands. Van de Meerendonk et al. [30] translated these two subscales and adapted them to a Dutch context from their original form by Herek [21, 32]. Both subscales consisted of five items and answer categories on a 5-point Likert scale, ranging from 1 (totally agree) to 5 (totally disagree). Examples of items included in the scales are: “sex between two women is not natural” and “gay men are just not real men.” All item formulations can be found in the supplementary material (S1 Table). Some items also needed minor simplification in terms of language, considering the younger age range of our participants. However, the changes to the items were minor and related to grammar only. Van de Meerendonk et al.’s [33] data support the scales’ acceptable reliability in the Dutch context for adults, for Attitudes Toward Lesbians (Cronbach’s *α* = .90) and Attitudes Toward Gay Men (*α* = .88).

#### Attitudes toward bisexual people

To measure students’ Attitudes Toward Bisexual People, we used an adapted version of the instrument used by Mulick and Wright [34] in their study on biphobia. The original scale consisted of 30 items and had an overall estimated reliability of *α* = .94. For this study, we shortened this scale so that the questionnaire would not become too long. We removed items that were not suitable for the Dutch context. The remaining items were reduced to five based on which ones were the most substantively inherent to the items from the Attitudes Toward Lesbians and Attitudes Toward Gay Men scales. By doing so, we selected five items that assess cognition, emotion and behavior that are relevant regarding measuring attitudes toward bisexual people. For uniformity reasons, we changed the answer categories from a 6-point Likert scale to a 5-point Likert scale, ranging from 1 (totally agree) to 5 (totally disagree). Using 5 instead of 6 categories has negligible effect on reliability and factorial validity of the scale [62, 63]. Examples of items are: “I avoid bisexual people” and “I think bisexuality is wrong.” The formulations of all items of the scale can be found in the supplementary material (S2 Table).

#### Statistical analysis plan

To examine the characteristics of the scales, the descriptive statistics of the mean scale score were calculated using the software program IBM SPSS Statistics 27.0 for Windows: The mean value, standard deviations, minimum and maximum of the mean scores for individual scales, and Pearson correlation between all pairs of mean scores were calculated. The differences between the mean scores of various categories can also be statistically tested. In many cases the standard deviations are smaller than 1. In this case, and with the sample sizes of this study, a mean difference of 0.14 between two categories (e.g., boys and girls) is also statistically significant at the 5% level. Furthermore, the reliability of each mean score was assessed with Cronbach’s alpha. We used the following criteria regarding the reliability: α ≥ .80 indicated high reliability, .70 ≤ α < .80 indicated moderate reliability and α < .70 indicated low reliability [64]. We used the following criteria for the strength of the correlations: *r* ≥.50 indicated a strong coherence, .30 ≤ *r* < .50 indicated a moderate coherence and *r* < .30 indicated a weak coherence [65].

**Fig 1:**
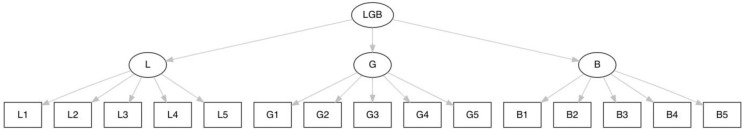
Second order factor model LGB.

The factorial validity of the scales was investigated with confirmatory factor analysis (CFA) using the statistical software program R (R Core Team, 2017) and the Lavaan package [66]. Factorial validity was first studied for each scale separately, since the scales were designed as separate dimensions. This was followed by considering a three-factor model (consisting of Attitudes Toward Lesbians, Gay Men and Bisexual People) and a second order model (see Fig 1) to test whether attitudes toward lesbians, gay men and bisexual people are underlying domains of a general attitude toward LGB people, for both Sample 1 and Sample 2. All model parameters were estimated using a diagonally weighted least squares (DWLS) estimation, which is suitable for ordinal variables. To assess model fit, we used the chi-squared statistic (χ^2^) and associated degrees of freedom, the comparative fit index (CFI), the Tucker-Lewis Index (TLI), the root mean squared error of approximation (RMSEA) and its 90% confidence interval (90% CI), and the standardized root mean squared residual (SRMR). Given the large sample sizes (N = 741 and N = 892), it was likely that all χ^2^-based model tests would be significant [67]. Thus, we focused primarily on the other fit measures. A threshold value of ≥ .90 for both the CFI and the TLI and of ≤ .10 for the RMSEA indicated a moderate fit. The cut-off points for the CFI and the TLI at ≥ .95 and for the RMSEA at ≤ .06 indicated a good fit. For the SRMR, the cut-off point for a good fit was set at ≤ .08 [68–70]. For an interpretation of the results, standardized coefficients and standard errors of the individual items and the latent constructs were used. For individual scales, if some statistic (TLI, CFI, RMSEA or SRMR) indicated that the associated model exhibited only a moderate model fit, modification indices were assessed to improve the model fit. An acceptable modification, and the only one applied in this study, is allowing a correlation between two items from the same scale if this is substantively meaningful [71, 72]. If adding such a correlation improves model fit sufficiently, the improvement indicates that the two items measure another construct, in addition to the construct assessed with all items of the scale.

Measurement invariance tests were performed to answer the second aim. For both gender (cisgender boys and cisgender girls) and education level (prevocational education, senior general and pre-university education), we considered three models, namely, a model of configural invariance (M1; multiple groups), weak or metric invariance (M2; multiple groups with equal factor loadings) and strong or scalar invariance (M3; multiple groups with equal factor loadings and equal intercepts) [73]. Models M1 to M3 are increasingly restrictive and nested. To decide on the tenability of the measurement model, we compared M3 with both M1 and M2, and, if necessary, M2 with M1 in order to get a good fit. If the difference between the CFI values (M1 –M3, or M2 –M3) was ≤ .01 and the difference in the RMSEA (M3 –M1, or M3 –M2) was ≤ .015, the more restrictive model was preferred [74, 75]. For the difference in the TLI, we used ≤ .01 as a threshold value. For all tests we used a significance level of .05.

## Results

### Descriptive statistics

Table 1 presents various descriptive statistics for Sample 1 (N = 741) and Sample 2 (N = 892), that is, the mean value (M), the standard deviation (SD), the minimum, the maximum and Cronbach’s alpha. For both Samples 1 and 2, the analyses indicate that students had, on average, relatively positive attitudes toward lesbians, gay men and bisexual people, since the mean values were 3.90–4.23 on a 5-point scale (score 5 equates to maximum positive). Students varied little in their range of answers (SD .86–1.05), but all answer options were chosen at least once (min = 1.00 and max = 5.00 for all scales).

**Table 1:** Descriptive statistics (mean, standard deviation, minimum, maximum and Cronbach’s alpha) for Samples 1 and 2.

**Sample 1**	**M**	**SD**	**Min**	**Max**	**alpha**
Attitude toward Lesbians	4.07	.86	1.00	5.00	.84
Attitude toward Gay Men	3.95	.97	1.00	5.00	.89
Attitude toward Bisexual People	4.23	.90	1.00	5.00	.92
**Sample 2**					
Attitude toward lesbians	3.99	.92	1.00	5.00	.85
Attitude toward Gay Men	3.90	1.05	1.00	5.00	.91
Attitude toward Bisexual People	4.09	.98	1.00	5.00	.95

The estimated reliability of all scales was moderate to high (> .70). In Sample 1, Cronbach’s alpha was the lowest for Attitude Toward Lesbians (α = .84), and in Sample 2, it was the highest for Attitude Toward Bisexual People (α = .95).

Tables 2–4 present descriptive statistics for, respectively, attitudes toward lesbians, gay men and bisexual people, across educational level and gender. The patterns in the three tables are very similar. For each attitude scale and both samples, pre-vocational education students had lower average scores than students in senior general and pre-university education. Furthermore, for each attitude scale, each educational level and both samples, the average score of girls was higher than the average score of boys. The main effect of gender on these attitudes (in terms of mean differences) is quite strong, even stronger than the main effect of educational level. Combining these two main effects demonstrates that girls in higher educational levels consistently had the most positive attitudes toward lesbians, gay men and bisexual people (M = 4.33–4.56), whereas boys in pre-vocational education had the least positive attitudes (M = 3.10–3.56). If we compare the three attitude scales, boys had their highest average scores on Attitude toward Bisexual people (M = 3.33–3.95) and their lowest average scores on Attitude toward Gay Men (M = 3.10–3.59). In addition, girls had their lowest average scores on Attitude toward Lesbians (M = 3.98–4.35), and just as for boys, the highest average scores on Attitude toward Bisexual people (M = 4.21–4.56).

**Table 2:** Attitude toward lesbians—comparison of descriptive statistics across educational level and gender.

**Sample 1**	M	SD	Min	Max	alpha
Pre-vocational education	3.75	.93	1.00	5.00	.80
*Boys*	*3*.*53*	.*77*	*1*.*00*	*5*.*00*	.*73*
*Girls*	*3*.*98*	*1*.*00*	*1*.*00*	*5*.*00*	.*84*
Senior general and pre-university education	4.14	.83	1.00	5.00	.84
*Boys*	*3*.*88*	.*86*	*1*.*00*	*5*.*00*	.*84*
*Girls*	*4*.*34*	.*75*	*1*.*00*	*5*.*00*	.*83*
**Sample 2**					
Pre-vocational education	3.75	.95	1.00	5.00	.83
*Boys*	*3*.*51*	.*93*	*1*.*00*	*5*.*00*	.*81*
*Girls*	*4*.*03*	.*85*	*1*.*00*	*5*.*00*	.*80*
Senior general and pre-university education	4.01	.89	1.00	5.00	.85
*Boys*	*3*.*72*	.*97*	*1*.*00*	*5*.*00*	.*86*
*Girls*	*4*.*35*	.*70*	*1*.*00*	*5*.*00*	.*78*

* p < .05

Since the prevalence of non-cisgender participants in both samples was statistically negligible, only cisgender boys and cisgender girls were included when generating descriptive statistics across gender.

**Table 3:** Attitude toward gay men—comparison of descriptive statistics across educational level and gender.

**Sample 1**	**M**	**SD**	**Min**	**Max**	**alpha**
Pre-vocational education	3.65	1.06	1.00	5.00	.90
*Boys*	*3*.*25*	.*96*	*1*.*00*	*5*.*00*	.*90*
*Girls*	*4*.*05*	.*96*	*1*.*00*	*5*.*00*	.*88*
Senior general and pre-university education	4.01	.94	1.00	5.00	.89
*Boys*	*3*.*59*	.*96*	*1*.*00*	*5*.*00*	.*87*
*Girls*	*4*.*33*	.*78*	*1*.*00*	*5*.*00*	.*86*
**Sample 2**					
Pre-vocational education	3.54	1.08	1.00	5.00	.90
*Boys*	*3*.*10*	*1*.*03*	*1*.*00*	*5*.*00*	.*88*
*Girls*	*4*.*11*	.*80*	*1*.*00*	*5*.*00*	.*83*
Senior general and pre-university education	4.07	.99	1.00	5.00	.91
*Boys*	*3*.*56*	*1*.*08*	*1*.*00*	*5*.*00*	.*92*
*Girls*	*4*.*43*	.*68*	*1*.*00*	*5*.*00*	.*83*

* p < .05

Since the prevalence of non-cisgender participants in both samples was statistically negligible, only cisgender boys and cisgender girls were included when generating descriptive statistics across gender.

**Table 4:** Attitude toward bisexual people—comparison of descriptive statistics across educational level and gender.

**Sample 1**	**M**	**SD**	**Min**	**Max**	**alpha**
Pre-vocational education	3.90	1.01	1.00	5.00	.92
*Boys*	*3*.*56*	.*98*	*1*.*00*	*5*.*00*	.*90*
*Girls*	*4*.*21*	.*92*	*1*.*00*	*5*.*00*	.*95*
Senior general and pre-university education	4.30	.86	1.00	5.00	.92
*Boys*	*3*.*95*	.*93*	*1*.*00*	*5*.*00*	.*91*
*Girls*	*4*.*56*	.*66*	*1*.*00*	*5*.*00*	.*91*
**Sample 2**					
Pre-vocational education	3.72	1.10	1.00	5.00	.95
*Boys*	*3*.*33*	*1*.*02*	*1*.*00*	*5*.*00*	.*93*
*Girls*	*4*.*23*	.*91*	*1*.*00*	*5*.*00*	.*96*
Senior general and pre-university education	4.26	.88	1.00	5.00	.95
*Boys*	*3*.*84*	.*97*	*1*.*00*	*5*.*00*	.*94*
*Girls*	*4*.*56*	.*64*	*1*.*00*	*5*.*00*	.*93*

* p < .05

Since the prevalence of non-cisgender participants in both samples was statistically negligible, only cisgender boys and cisgender girls were included when generating descriptive statistics across gender.

Boys seemed to have varied more in their range of answers than girls did. Especially in the academic tracks, boys seemed to differ substantially in their attitudes toward lesbian, gay men and bisexual people (SD = .96–1.08). Nevertheless, in each category of scale, sample, educational level and gender, all answer options of the 5-point Likert scale were used at least once. Descriptive statistics per individual item and frequency distributions for all scores on the 5-point Likert scale can be found in the supplementary material (S2 Table). For some items there seems to be a ceiling effect.

### Correlations

Table 5 presents the correlation coefficients between the attitude scales (attitude toward lesbians, attitude toward gay men and attitude toward bisexual people) for Samples 1 and 2. We found strong positive correlations between the three attitude scales for both Sample 1 (*r* = .78–.83) and Sample 2 (*r* = .79–.85) and significant (*p* < .05) correlations between Attitude toward Lesbians, Attitude toward Gay Men and attitude toward Bisexual People. This indicates that students with a positive attitude toward lesbians were also more positive toward gay men and bisexual people, and vice versa.

**Table 5:** Correlation coefficients between the attitude scales for Samples 1 and 2.

**Sample 1**	**1**	**2**	**3**
1. Attitude toward Lesbians	1		
2. Attitude toward Gay Men	.83*	1	
3. Attitude toward Bisexual People	.79*	.78*	1
**Sample 2**	**1**	**2**	**3**
1. Attitude toward Lesbians	1		
2. Attitude toward Gay Men	.80*	1	
3. Attitude toward Bisexual People	.79*	.85*	1

* p < .05

### Confirmatory factor analyses

Table 6 presents the fit indices for the CFA models for Sample 1. The model fit is good for attitude toward lesbians (CFI = .999, TLI = .997, RMSEA = .055, SRMR = .025), the three-factor model (CFI = .999, TLI = .998, RMSEA = .055, SRMR = .032) and the second-order model (CFI = .999, TLI = .998, RMSEA = .056, SRMR = .032). For attitude toward gay men and bisexual people, the model fit is good in terms of CFI (.999), TLI (.998 and .985, respectively) and SRMR (.022 and .023, respectively), but moderate in terms of RMSEA (.061 and .082, respectively). For attitude toward bisexual people, the modification indices indicated that allowing an additional correlation between items 1 and 2 of the scale would make the biggest improvement to the model fit. Indeed, the row of Table 4 corresponding to Attitude B* shows that, after this modification was applied, the model fit for attitude toward bisexual people was excellent (CFI = TLI = 1.000, RMSEA = .022, SRMR = .012).

**Table 6:** Goodness-of-fit indices for factor models for Sample 1.

	χ2 (df)	CFI	TLI	RMSEA [90% CI]	SRMR
Attitude toward Lesbians	16.280 (5)	.999	.997	.055 [.027, .086]	.025
Attitude toward Gay Men	18.966 (5)	.999	.998	.061 [.034, .092]	.022
Attitude toward Bisexual People	29.952 (5)	.999	.985	.082 [.055, .112]	.023
Attitude toward Bisexual People*	8.534 (4)	1.000	1.000	.039 [.000, .076]	.012
3-factor model	284.395 (87)	.999	.998	.055 [.048, .063]	.032
2^nd^ order model	283.970 (86)	.999	.998	.056 [.049, .063]	.032

* Denotes the modified model.

The good model fit for all Sample 1 models provides evidence that attitudes toward lesbians, gay men and bisexual people can be measured reliably among Dutch eighth- and ninth-graders. Furthermore, the estimated correlations between the latent variables of the three-factor model are .91, .92 and .98 These high correlations suggest that, for Dutch eighth- and ninth-graders, attitudes toward lesbians, gay men and bisexual people may be underlying domains of a general attitude toward LGB people. Further evidence for this hypothesis is provided by the good model fit associated with the second-order model.

Table 7 presents the fit indices for the CFA models for Sample 2. The model fit is good for attitude toward gay men (CFI = TLI = 1.00, RMSEA = .000, SRMR = .009), the three-factor model and the second-order model (both CFI = TLI = .999, RMSEA = .050, SRMR = .024. For attitude toward lesbians and bisexual people, the model fit is good in terms of the CFI, the TLI and the SRMR, but moderate or below moderate in terms of the RMSEA. When adding a correlation between items 2 and 4 of attitude toward lesbians, the model fit becomes good (RMSEA = .030). Analogous to the Sample 1 model for attitude toward bisexual people, adding a correlation between corresponding items 4 and 5 improved the model fit to good (RMSEA = .040).

**Table 7:** Goodness-of-fit indices for factor models for Sample 2.

	χ2 (df)	CFI	TLI	RMSEA [90% CI]	SRMR
Attitude toward Lesbians	35.012 (5)	.998	.996	.082 [.058, .109]	.029
Attitude toward Lesbians*	7.301 (4)	1.000	.999	.030 [.000, .065]	.015
Attitude toward Gay Men	4.559 (5)	1.000	1.000	.000 [.000, .044]	.009
Attitude toward Bisexual People	50.785 (5)	1.000	.999	.101 [.077, .128]	.018
Attitude toward Bisexual People*	9.662 (4)	1.000	1.000	.040 [.005, .073]	.008
3-factor model	278.346 (87)	.999	.999	.050 [.043, .056]	.024
2^nd^ order model	278.346 (87)	.999	.999	.050 [.043, .056]	.024

* Denotes modified models.

Table 8 presents the measurement invariance tests with regard to educational level for both Samples 1 and 2. We first considered the CFA models for Sample 1. For all three scales (attitudes toward lesbians, gay men and bisexual people (modified)), the more restrictive model M3 actually has a better model fit than models M1 and M2 in terms of the CFI, the TLI and the RMSEA. All differences with regard to the CFI, the TLI and the RMSEA between M1 and M2, on the one hand, and M3, on the other hand, are thus negative, and smaller than the threshold values. Hence, these numbers provide ample evidence that the same constructs (attitudes toward lesbians, gay men and bisexual people) were measured across educational levels.

**Table 8:** Measurement invariance tests for education level.

Sample 1	χ2 (df)	CFI	TLI	RMSEA [90% CI]	SRMR
Attitude toward Lesbians–M1	19.245 (15)	.999	.999	.034 [.000, .073]	.028
Attitude toward Lesbians–M2	37.366 (23)	.998	.998	.050 [.016, .079]	.037
Attitude toward Lesbians–M3	48.812 (51)	1.000	1.000	.000 [.000, .038]	.031
Attitude toward Gay Men–M1	23.122 (15)	1.000	.999	.047 [.000, .083]	.024
Attitude toward Gay Men–M2	41.268 (23)	.999	.999	.057 [.027, .084]	.031
Attitude toward Gay Men–M3	56.104 (51)	1.000	1.000	.020 [.000, .047]	.024
Attitude toward Bisexual People–M1*	20.110 (12)	1.000	.999	.052 [.000, .091]	.017
Attitude toward Bisexual People–M2*	34.685 (20)	.999	.999	.055 [.021, .084]	.026
Attitude toward Bisexual People–M3*	44.689 (48)	1.000	1.000	.000 [.000, .037]	.018
**Sample 2**					
Attitude toward Lesbians–M1	33.517 (15)	.999	.997	.065 [.035, .094]	.029
Attitude toward Lesbians–M2	55.709 (23)	.998	.997	.069 [.046, .093]	.036
Attitude toward Lesbians–M3	81.218 (51)	.998	.999	.045 [.025, .062]	.029
Attitude toward Gay Men–M1	8.188 (15)	1.000	1.000	.000 [.000, .020]	.012
Attitude toward Gay Men–M2	84.258 (23)	.999	.998	.095 [.074, .117]	.039
Attitude toward Gay Men–M3	64.555 (51)	1.000	1.000	.030 [.000, .050]	.016
Attitude toward Bisexual People–M1*	33.517 (15)	.999	.997	.065 [.035, .094]	.029
Attitude toward Bisexual People–M2*	85.898 (20)	.999	.999	.105 [.083, .129]	.022
Attitude toward Bisexual People–M3*	44.199 (48)	1.000	1.000	.000 [.000, .033]	.011

* Denotes modified models.

The numbers presented in Table 9 correspond to Sample 2: For attitudes toward lesbians and bisexual people (modified), the more restrictive model M3 has a better model fit than models M1 and M2 in terms of the CFI, the TLI and the RMSEA. Thus, these numbers indicate that the same constructs were measured across educational levels. For attitude toward gay men only, the difference in the RMSEA values is above the threshold value when comparing M3 and M1 (.030 –.000 = .030 ≥ .015).

**Table 9:** Measurement invariance tests for gender.

Sample 1	χ2 (df)	CFI	TLI	RMSEA [90% CI]	SRMR
Attitude toward Lesbians–M1	15.677 (10)	.999	.998	.040 [.000, .075]	.025
Attitude toward Lesbians–M2	36.872 (14)	.997	.996	.067 [.041, .094]	.040
Attitude toward Lesbians–M3	51.286 (28)	.997	.998	.048 [.026, .068]	.028
Attitude toward Gay Men–M1	23.833 (10)	.999	.998	.062 [.030, .094]	.026
Attitude toward Gay Men–M2	34.407 (14)	.999	.998	.063 [.037, .091]	.034
Attitude toward Gay Men–M3	43.133 (28)	.999	.999	.039 [.011, .060]	.028
Attitude toward Bisexual People–M1*	12.233 (8)	1.000	1.000	.038 [.000, .078]	.015
Attitude toward Bisexual People–M2*	23.239 (12)	1.000	.999	.051 [.017, .082]	.023
Attitude toward Bisexual People–M3*	43.217 (26)	.999	.999	.043 [.018, .065]	.017
**Sample 2**					
Attitude toward Lesbians–M1	47.862 (10)	.997	.993	.095 [.069, .123]	.036
Attitude toward Lesbians–M2	65.816 (14)	.995	.993	.094 [.072, .118]	.041
Attitude toward Lesbians–M3	87.578 (28)	.995	.996	.071 [.055, .089]	.038
Attitude toward Gay Men–M1	5.991 (10)	1.000	1.000	.000 [.000, .032]	.011
Attitude toward Gay Men–M2	17.266 (14)	1.000	1.000	.024 [.000, .056]	.019
Attitude toward Gay Men–M3	26.882 (28)	1.000	1.000	.000 [.000, .036]	.013
Attitude toward Bisexual People–M1*	12.943 (8)	1.000	1.000	.038 [.000, .075]	.010
Attitude toward Bisexual People–M2*	27.171 (12)	1.000	1.000	.055 [.027, .083]	.016
Attitude toward Bisexual People–M3*	25.307 (26)	1.000	1.000	.000 [.000, .037]	.010

* Denotes modified models.

Table 9 presents the measurement invariance tests with regard to gender for both Samples 1 and 2. For all the CFA models of Sample 1, it holds that differences with regard to the CFI, the TLI and the RMSEA between M1 and M2, on the one hand, and M3, on the other hand, are smaller than the threshold values. Hence, these Sample 1 numbers provide ample evidence that the same constructs (attitudes toward lesbians, gay men and bisexual people) were measured across gender.

The numbers presented in Table 9 correspond to Sample 2. For the three attitude scales, the more restrictive model M3 actually has a better model fit than models M1 and M2 in terms of the CFI, the TLI and the RMSEA. Again, this provides ample evidence that the same constructs were measured across gender. The differences in the CFI, the TLI and the RMSEA between M3, on the one hand, and M1 and M2, on the other hand, are also above the threshold values.

**Fig 2:**
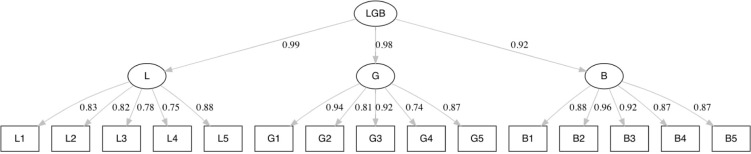
Second order model LGB with estimated factor loadings based on Sample 1.

**Fig 3:**
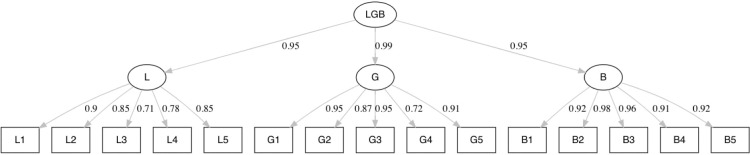
Second order model LGB with estimated factor loadings based on Sample 2.

Fig 2 presents the model with the estimated factor loadings and associated standard errors (between brackets) between the observed and latent variables for Sample 1. All factor loadings in Fig 1 are significant at the .001 level. The same applies to Fig 3 in respect of Sample 2.

## Discussion

The first aim of this study was to test the factorial validity and reliability of adapted instruments of attitudes toward LGB persons, since the psychometric properties have not been studied previously among adolescents in secondary schools in the Dutch context. The estimated reliability of all scales was moderate (≥ .70) to high. The model fit statistics in Tables 6 and 7 provide ample evidence for the factorial validity of the three instruments and associated constructs. Only minor model modifications were needed to obtain good to excellent model fit. In other words, evidence was provided that the three constructs of attitudes toward lesbians, gay men and bisexual people can be measured quite well among Dutch eighth- and ninth-graders.

The second aim of this study was to test the measurement invariance of the adapted instruments across gender and educational level. The model fit statistics provide ample evidence for strong measurement invariance, that is, evidence that the same constructs (attitudes toward lesbians, gay men and bisexual people) were measured across educational level and gender among Dutch adolescents. Thus, these measures are adequate for adolescents regardless of gender and whether they are enrolled in vocational or academic educational tracks.

Since stigma against LGB people continues to be widespread among adolescents and previous research in the Netherlands has been compromised by the use of single items as sexual diversity measure, the third aim of this study was to examine Dutch early adolescents’ attitudes toward lesbians, gay men and bisexual people using scales. We did so among eighth- and ninth-graders in two samples (N = 741 and N = 892). We found that overall, participants hold on average relatively positive attitudes toward LGB people. The results are in line with previous research regarding the Netherlands being a relatively affirmative context in regard to sexual diversity, also among younger people [24, 30]. However, medium attitudes were also found in subgroups. Educational level consistently impacted the mean differences in attitudes. Students in pre-vocational training had on average less favorable attitudes. Previous research showed that young adolescents in pre-vocational education feel less safe in their school as students in senior general or pre-university education. This is among other things related to a higher prevalence of bullying in lower levels of education [76, 77]. From our study, we can hypothesize that decreased LGB safety in pre-vocational tracks and/or schools may also be related to less optimal attitudes towards LGB people. In addition, our findings might also be related to socialization. Students in pre-vocational education more often have lower educated parents than students in more academic educational tracks [78]. Research shows that lower educated people are less likely to agree with equal rights for sexual minorities and that parental attitudes on this topic are transmittedto children, which could explain that we found less positive attitudes for this group. Implications for practice could concern that adolescents in pre-vocational education may need a different approach regarding interventions on sexual diversity than students who are in more academic tracks [78].

Gender also had a strong effect regarding attitudes toward LGB people. We found, corresponding to other studies’ empirical results [20, 53, 54], that girls across all educational levels hold more affirmative attitudes toward lesbians, gay men and bisexual people. Thus, even though attitudes of Dutch youth toward sexual diversity have become more positive over the years [24, 30], it seems to be the case that there are large differences between boys and girls. Our results are in line with other scientific studies among Western populations, indicating that boys especially are more negative toward gay men in comparison to lesbians and bisexual people [20, 25, 26, 30]. Even though, gender and educational level have strong effects on attitudes toward LGB as an overarching scale, we found that boys are generally a bit more negative toward gay men in particular. In addition, previous research shows that bisexual people experience a ‘double stigma’ and report lower levels on wellbeing than lesbians and gay men [37, 38, 79]. However, in our study, the most positive attitudes were found for bisexual people. This applied for both boys and girls across different educational levels. This leads to implications for further research regarding whether the position of bisexual people is extra precarious among young adolescents in modern times and to which factors this might be related. However, we measured attitudes of individual young adolescents toward bisexual people, which is related to only one source of minority stress for this population. Future studies should include scales on more factors regarding biphobia and bisexual people’s wellbeing in order to thoroughly examine the position of bisexual individuals.

The fourth aim of this study was to explore relationships between attitudes toward lesbians, attitudes toward gay men and attitudes toward bisexual people for Dutch eighth- and ninth-graders. The correlations between the three attitude scales were very high, and all correlations were significant (*p* < .05). These results indicate that whenever students had positive attitudes to lesbians, they tended to show positive attitudes toward gay man and bisexual people as well, and vice versa. These findings do not align with predominant international conclusions regarding that people tend to significantly hold less affirmative attitudes toward gay men as toward lesbians [20, 24–26]. Multiple studies show that gender identity influences people’s attitudes toward lesbian women in comparison to gay men. For instance, in Western contexts, male adolescents often hold significantly more negative attitudes toward gay men than to lesbians. This disparity is possibly due to the sexualization of lesbian women and prevailing gender norms that particularly bias against gay men [20, 22, 24–26].

Subsequently, we aimed to contribute to the empirical debate about whether, for adolescents, attitudes toward lesbians, gay men and bisexual people can be understood as an underlying domain of a general attitude toward LGB people. The good fits of the second-order factor models indicate that Attitude Toward Lesbian Women, Gay Men and Bisexual People is associated with an overarching LGB construct. Even though, we found small mean differences in boys’ attitudes (boys overall are slightly more negative toward gay men than toward lesbians or bisexual people), the high correlations between the different attitudes and the good fit of second order factor models provide evidence that Dutch students do not differentiate much in how they think and evaluate homosexuality, lesbianism and bisexuality. This differs from previous research that indicated that students differentiate substantially in terms of their attitudes toward gay men, lesbian women [13, 14, 21] and bisexual people [37]. When one wants to examine moderators or trends (e.g., boys and adolescents in lower levels of education hold a less affirmative attitude toward LGB people) it suffices to measure one construct or use one general measurement instead of examining all three constructs separately. Possibly, this goes to show that young people in current Dutch society are highly familiar with non-heterosexuality as a phenomenon. The prevalence of youth identifying as LGB is increasing, and the age at which youth first come out is decreasing, which is likely driven by younger generations such as “Generation Z” [80, 81]. The increased visibility of sexual and gender minorities in Western Europe (including on social media) may highlight important new directions in the ways that younger generations think about sexual minority identities [82]. Furthermore, the introduction of a law for compulsory education on the topic of sexual and gender diversity in the Netherlands (since 2012) [83] has probably familiarized students with non-heterosexual people and possibly contributed to unified, similar perceptions of various sexual minorities. Human sexual behaviors follow a type of social script (‘Sexual script theory’) [84]. This societal familiarization of younger generations with sexual diversity might have an impact on adolescents’ ‘sexual scrips’ and thus also on how we perceive sexualities. A counterhypothesis would be that early adolescents, also in the Netherlands, still have relatively limited knowledge about various sexually diverse subgroups, and this may explain their highly similar attitudes to lesbians, gay men and bisexual people [85]. Nevertheless, the scientific evidence of young people’s increased awareness of sexuality and diversity due to the internet and social media over the past decades, as well as the compulsory education on sexual diversity, render our counterhypothesis less plausible.

Even though an overarching image can be drawn without differentiating between sexual orientations, we advise researchers that are interested in particular attitudes to still measure attitudes toward LGB with separate constructs since subtle mean differences may still be found (in our case: a slightly more negative attitude toward gay men among male young adolescents in a relatively tolerant Western context).

### Methodological considerations and limitations

Future researchers may want to consider using one scale to measure students’ attitude toward LGB instead of separating the scales into three constructs. However, in order to confirm whether modern young people no longer differentiate much between lesbian women, gay men and bisexual people (and thus hold similar attitudes toward them), further research in other cultural contexts is recommended. Also, past studies have indicated that the position of bisexual people is especially precarious, and that they experience a double stigma [37, 79]. The results of the current study suggest that young adolescents are not more negative toward bisexual people when they hold positive attitudes toward lesbians and gay men. Further research might explore whether the prevalence of biphobia has decreased. In addition, with regard to the ‘double stigma’ subgroups should be identified. Thus, examining whether gay or lesbian populations hold more negative attitudes toward bisexual people specifically.

The samples used in this study are likely to be representative for the population of Dutch pupils in the eighth and ninth grades (based on the proportionality of different demographics as gender, religion, age and ethnicity), since they consisted of different educational levels from all regions (both urban and rural) of the Netherlands and all were state-funded schools (some with Christian foundations/convictions but mostly non-religious). Nevertheless, the results should be interpreted with some caution. Participating in the LGB reading intervention in Dutch literature classes was based on schools’ and teachers’ choice and self-registration and thus, presumably, a positive interest in, or a sense of urgency around, improving the class climate around sexual diversity. A rather positive view with regard to sexual diversity may have an effect on the overall representativeness of the data. On the other hand, it is plausible that our results are influenced by socially desirable answering. In addition, data was collected during the COVID pandemic. As mentioned, we used two separate samples, as they were collected in different years that the pandemic was present and policies tended to change. For instance, at certain moments schools were partially closed or varying rules of conduced were (temporarily) obtained in classrooms. Interestingly, we found only small differences between the samples.

Furthermore, we found discrepancies in our results in comparison to studies that focus on the international population which might need further investigation. Various international studies indicate that people tend to have significant more negative attitudes toward gay men than toward lesbians [20, 24–26]. Our results do not provide strong evidence for this tendency. Thus, for the generalizability of our results regarding the suitability of the instruments in the Dutch context, and that Dutch young adolescents do not differentiate much regarding their attitudes toward lesbians, gay men and bisexual people, future studies should conduct further tests. Lastly, the focus of this study is on psychometric properties of the used instrument. Considering the large sample size, we were able to provide some basic information on Dutch young adolescents’ attitudes toward sexual diversity based on descriptive statistics and correlations. However, more elaborate statistical testing is needed in order to provide strong conclusions on this topic. Also, we took factors as gender and educational level into account, but there are different social and demographic differences and moderators that affect people’s attitudes toward sexual diversity that should be examined in future research. For example, age seems of relevance; younger adolescents (12–14 years old) hold more affirmative attitudes toward LGB people than older adolescents (15+ years old). The same applies to adolescents who have LGB friends in comparison to those who experience limited or have no contact with LGB people [52].

Some methodological challenges emerged. For Attitude Toward Lesbians and Attitude Toward Bisexual People, one modification of the confirmatory factor analysis (CFA) models, adding a correlation between two items of the scale, was needed to achieve an adequate model fit: In addition to the general construct of attitude toward lesbians, two items appeared to assess a second construct. For Attitude Toward Lesbians, the corresponding two items consider sexual behavior between two women, whereas the other items consider participants’ opinion on lesbian women in general and their presence in society, with item 2 stating “Sex between two women is not natural” and item 4 stating “Sex between two lesbian women is disgusting.” The results indicated that these items are conceptually similar. For the construct of Attitude Toward Bisexual People, a similar situation occurred. Two of the five items appeared to assess a second construct. The corresponding two items consider that participants have to imagine interacting with bisexual people or being around them, whereas the other items aggregate participants’ general opinions about bisexuality, with item 4 stating “I feel uneasy around bisexual people” and item 5 stating “I would not go to a public place where I knew there would be bisexual individuals.” These items are also conceptually similar, possibly because they both refer to a physical proximity to bisexual people.

In conclusion, the measurements by Herek [16, 17, 21] (and adapations by Van Meerendonk [33]) as well Muclick and Wrirght [31] used prove to be psychometrically sound among Dutch young adolescents who grow up at present. In addition, we found that attitudes toward sexual diversity are overall affirmative among Dutch youth, while gender and educational level played a predictive role in this respect. Dutch young adolescents do not seem to distinguish much between Lesbian, Gay and Bisexual people, which possibly relates back to the umbrella of LGBTQI+ as a label that is currenty increasingly common. future research to.

## Supporting information

S1 Table Item formulations. (DOCX)

S2 Table Descriptive statistics per individual item and frequency distribution for scores 1–5. (DOCX)
