# Supplementary material for: Assessing attitudes toward LGB people in young adolescents
Source: PLoS One. 2024 Oct 8;19(10):e0305057. doi: 10.1371/journal.pone.0305057 (PMC11460697; doi:10.1371/journal.pone.0305057)
Supplement: S1 Table — (DOCX) [file pone.0305057.s001.docx]

S1 Table. Item formulations

| Atittudes toward Lesbians | Item 1 (L1) | “Lesbian sexuality just does not fit in our society” |
| --- | --- | --- |
|  | Item 2 (L2) | “Sex between two women is not natural” |
|  | Item 3 (L3) | “Lesbian sexuality is not a problem for me” |
|  | Item 4 (L4) | “Sex between two women is disgusting” |
|  | Item 5 (L5) | “Lesbian women are abnormal” |
| Attitude toward Gay Men | Item 1 (G1) | “I disapprove of male homosexuality” |
|  | Item 2 (G2) | “Homosexual men are just not real men” |
|  | Item 3 (G3) | “Sex between two men is just plain wrong” |
|  | Item 4 (G4) | “Male homosexuality is a natural expression of sexuality in men” |
|  | Item 5 (G5) | “Male homosexuality goes against human nature” |
| Attitude toward Bisexual People | Item 1 (B1) | “I do not like bisexual individuals” |
|  | Item 2 (B2) | “I think bisexuality is wrong” |
|  | Item 3 (B3) | “I avoid bisexual people” |
|  | Item 4 (B4) | “I feel uneasy around bisexual people” |
|  | Item 5 (B5) | “I would not go to a public place where I knew there would be bisexual individuals” |
