# Supplementary material for: Assessing attitudes toward LGB people in young adolescents
Source: PLoS One. 2024 Oct 8;19(10):e0305057. doi: 10.1371/journal.pone.0305057 (PMC11460697; doi:10.1371/journal.pone.0305057)
Supplement: S2 Table — (DOCX) [file pone.0305057.s002.docx]

S2 Table. Descriptive statistics per individual item and frequency distribution for scores 1-5

| **Sample 1** |  |  |  |  |  |  |  |  |
| --- | --- | --- | --- | --- | --- | --- | --- | --- |
| **Scale** | **Item** | **M** | **SD** | **1** | **2** | **3** | **4** | **5** |
| **Attitude toward Lesbians** | L1 - Lesbian sexuality just does not fit in our society | 4.27 | 1.08 | 3.9% | 4.7% | 10.4% | 22.4% | 58.4% |
|  | L2 - Sex between two women is not natural | 3.79 | 1.22 | 6.5% | 8.5% | 22.0% | 24.9% | 37.7% |
|  | L3 - Lesbian sexuality is not a problem for me* | 4.28 | 1.05 | 4.2% | 3.1% | 10.9% | 24.3% | 57.3% |
|  | L4 - Sex between two lesbian women is disgusting | 3.68 | 1.22 | 6.6% | 9.2% | 28.4% | 20.9% | 34.6% |
|  | L5 - Lesbian women are abnormal | 4.35 | .93 | 2.6% | 2.0% | 10.6% | 27.0% | 57.5% |
| **Attitude toward Gay Men** | G1 - I disapprove of male homosexuality | 4.31 | 1.00 | 3.0% | 2.8% | 12.4% | 24.1% | 57.4% |
|  | G2 - Homosexual men are just not real men | 4.08 | 1.17 | 5.5% | 5.5% | 14.2% | 24.8% | 49.7% |
|  | G3 - Sex between two men is just plain wrong | 3.94 | 1.21 | 6.2% | 7.0% | 17.3% | 25.1% | 44.2% |
|  | G4 - Male homosexuality is a natural expression of sexuality in men* | 3.54 | 1.24 | 8.1% | 10.9% | 27.9% | 24.4% | 28.4% |
|  | G5 - Male homosexuality goes against human nature | 3.86 | 1.19 | 5.1% | 9.2% | 19.8% | 25.9% | 39.8% |
| **Attitude toward Bisexual People** | B1 - I do not like bisexual individuals | 4.15 | 1.05 | 2.8% | 4.6% | 17.9% | 23.5% | 50.9% |
|  | B2 – I think bisexuality is wrong | 4.29 | 1.00 | 2.8% | 3.5% | 12.4% | 24.1% | 56.9% |
|  | B3 – I avoid bisexual people | 4.33 | .97 | 2.8% | 2.6% | 11.6% | 24.8% | 58.0% |
|  | B4 – I feel uneasy around bisexual people | 4.17 | 1.04 | 2.8% | 3.9% | 17.8% | 23.7% | 51.5% |
|  | B5 – I would not go to a public place where I knew there would be bisexual individuals | 4.22 | 1.05 | 3.4% | 4.3% | 13.5% | 24.5% | 54.0% |
| **Sample 2** |  |  |  |  |  |  |  |  |
| **Scale** | **Item** | **M** | **SD** | **1** | **2** | **3** | **4** | **5** |
| **Attitude toward Lesbians** | L1 - Lesbian sexuality just does not fit in our society | 4.21 | 1.08 | 4.0% | 4.0% | 13.6% | 23.5% | 54.8% |
|  | L2 - Sex between two women is not natural | 3.81 | 1.27 | 6.7% | 10.8% | 18.5% | 22.8% | 41.3% |
|  | L3 - Lesbian sexuality is not a problem for me* | 3.99 | 1.23 | 7.7% | 4.8% | 15.2% | 25.4% | 46.7% |
|  | L4 - Sex between two lesbian women is disgusting | 3.74 | 1.24 | 6.2% | 10.5% | 24.0% | 21.4% | 37.9% |
|  | L5 - Lesbian women are abnormal | 4.18 | 1.05 | 3.6% | 3.1% | 16.3% | 25.8% | 51.2% |
| **Attitude toward Gay Men** | G1 - I disapprove of male homosexuality | 4.15 | 1.11 | 4.3% | 5.2% | 14.5% | 23.5% | 52.6% |
|  | G2 - Homosexual men are just not real men | 4.00 | 1.17 | 5.2% | 6.5% | 17.7% | 24.0% | 46.6% |
|  | G3 - Sex between two men is just plain wrong | 3.89 | 1.25 | 7.4% | 7.2% | 18.8% | 22.6% | 43.9% |
|  | G4 - Male homosexuality is a natural expression of sexuality in men* | 3.60 | 1.31 | 9.8% | 9.9% | 25.8% | 19.7% | 34.9% |
|  | G5 - Male homosexuality goes against human nature | 3.84 | 1.23 | 6.3% | 8.3% | 22.3% | 21.3% | 41.8% |
| **Attitude toward Bisexual People** | B1 - I do not like bisexual individuals | 4.03 | 1.10 | 3.7% | 5.0% | 22.0% | 22.9% | 46.4% |
|  | B2 – I think bisexuality is wrong | 4.16 | 1.02 | 2.8% | 3.5% | 17.7% | 26.7% | 49.3% |
|  | B3 – I avoid bisexual people | 4.14 | 1.04 | 2.7% | 4.0% | 19.4% | 24.2% | 49.7% |
|  | B4 – I feel uneasy around bisexual people | 4.04 | 1.08 | 2.6% | 7.2% | 19.7% | 24.6% | 46.0% |
|  | B5 – I would not go to a public place where I knew there would be bisexual individuals | 4.05 | 1.11 | 3.6% | 6.3% | 18.6% | 24.3% | 47.2% |

(1: strongly agree and 5: strongly disagree)

*Reversed Code Scale Items
